# Sleep Quality and Duration in European Adolescents (The AdolesHealth Study): A Cross-Sectional, Quantitative Study

**DOI:** 10.3390/children8030188

**Published:** 2021-03-03

**Authors:** Pablo Galan-Lopez, Raúl Domínguez, Thordis Gísladóttir, Antonio J. Sánchez-Oliver, Maret Pihu, Francis Ries, Markos Klonizakis

**Affiliations:** 1 Department of Communication and Education, Universidad Loyola Andalucía, 41704 Dos Hermanas (Sevilla), Spain; pgalan@uloyola.es; 2 Physical Education and Sports, Faculty of Educational Sciences, University of Seville, 41013 Sevilla (Sevilla), Spain; raul_dominguez_herrera@hotmail.com (R.D.); fries@us.es (F.R.); 3 Research Center for Sport and Health Sciences, School of Education, University of Iceland, 105 Reykjavík, Iceland; thg@hi.is; 4 Motricidad Humana y Rendimiento Deportivo, Universidad de Sevilla, 41013 Sevilla (Sevilla), Spain; 5 Institute of Sport Sciences and Physiotherapy, Faculty of Medicine, University of Tartu, 51005 Tartu, Estonia; maret.pihu@ut.ee; 6 Lifestyle, Exercise and Nutrition Improvement (LENI) Research Group, Department of Nursing and Midwifery, College of Health, Wellbeing and Life Sciences, Sheffield Hallam University, Sheffield S1 1WB, UK; klonizakis@gmail.com

**Keywords:** sleep quality, sleep duration, adolescents, healthy lifestyle

## Abstract

Sleep is a vital element of adolescents’ overall health; it influences their body and mind and thus affects their quality of life. Adequate sleep quality and duration are essential for maintaining optimal metabolic health and lowering the risk of developing several medical conditions, such as cardiovascular disease. The current study aimed to assess the perceived sleep quality and duration of 1717 European adolescents from three different European countries (Spain, Iceland and Estonia) aged 13- to 16-years (900 boys, 817 girls) using the Pittsburgh Sleep Quality Index (PSQI). A multivariate analysis of variance (MANOVA) was performed to examine differences between groups and two-factor analysis of variance (ANOVA) was used to analyze city and age differences. The probability of having poor sleep quality and duration was calculated by Odd-Ratio (OR). Our study found poor sleep quality in 44% of the boys and 53% of the girls, whereas 68% and 69%, respectively did not get the recommended hours of sleep (i.e., 8–10 h). No difference was found between adolescents from Estonia, Iceland and Spain regarding sleep duration. In contrast, Spanish and Estonian adolescents reported higher probabilities of having poor sleep quality. Finally, girls had a significantly higher probability of poor sleep quality than boys.

## 1. Introduction

Sleep is a recurrent phase, characterized by altered consciousness, relatively inhibited sensory activity and the inhibition of voluntary muscle activation, as well as impaired consciousness (REM phase) [1]. Optimal quality and duration of sleep are essential for appropriate development and physical growth in children and adolescents [2]. On the other hand, insufficient duration and low quality of sleep in early life are related to poor cognitive function and reduced academic performance with low attention and daytime concentration [3,4], as well as depressive symptoms [5]. 

Several studies have demonstrated that a lack of sleep increases the possibility of gaining weight and suffering from obesity due to different mechanisms that affect food intake and energy expenditure [6,7]. Additionally, good sleep quality is known to be essential for maintaining correct metabolic health and lower cardiovascular disease risk [8]. Furthermore, sufficient duration and good quality of sleep are directly related to a regular body composition index, less depressive symptoms and better sports performance [9]. Adequate sleep quality also ensures a positive emotional, physical and cognitive balance, and even correct physical recovery, reducing the risk of over-training and improving the rest and post-exercise recovery processes [10,11,12].

Adolescence is characterized by a clear decrease in the quality and duration of sleep of individuals [13,14,15]. Sleep disorders in adolescents have received significant attention due to their high prevalence and their negative effects on health outcomes [16]. Cross-sectional studies conducted on adolescent populations report sleep disorders and deterioration of daytime functions due to poor sleep quality or insufficient hours of sleep [17]. Combining the current tendency of late bedtime with not getting enough sleep due to early school hours and screen time exposure before bedtime and before sleep onset [18], reduces significantly the opportunity to obtain enough sleep of adequate quality [19]. Numerous studies on adolescents have shown that their sleep duration is insufficient, with an average of 7.5 h per day [20,21,22], when the recommended duration by reputable organizations (i.e., American Academy of Sleep Medicine) stands between 8–10 h [23,24]. 

Several studies have analyzed both duration and quality of sleep in adolescents. For example, Bel et al. (2013) studied the sleep duration of 1522 adolescents between 12 and 17 years. In this study, only 29% of the participants met the recommendations for daily sleep hours [25]. Similarly, a study carried out by Carvalho and colleagues (2019) showed perturbing results, as more than 39% of the participants presented poor sleep quality [26]. The authors concluded that those with an optimal sleep quality had a better quality of life (e.g., better nutrition and regular physical activity patterns). Another study on sleep quality in adolescents [27], determined that only 47% of the subjects reported duration of sleep following recommendations and that, in addition, those who slept less had higher rates of overweight or obesity. Numerous studies agree that low quality and short duration of sleep are directly related to bad dietary behaviors, low levels of physical activity and unhealthy habits, resulting in reduced quality of life [28,29,30].

Adolescence is a crucial period of life for the modelling of healthy habits that will persist in adulthood [31,32]. Furthermore, few studies on sleep duration and quality compare these variable with the intention to determine possible associations concerning such healthy habits [33]. Among the variables that influence sleep quality and quantity, we include age, gender, cultural aspects, and time habits. The published literature shows that adolescents tend to sleep less as they grow older and, evermore, there seems to be a significant difference in the sleep duration between school days and weekends [34]. In addition, the study of existing the literature reveals that there are differences in sleep duration and quality in relation to sex of the adolescents. Cultural aspects such as differences in meal times, especially at dinner [35], as well as geographical location have shown that people living in the western part of a time zone have both later bedtimes and shorter sleep duration than people in the eastern area of a time zone [36]. Besides, different geographical locations of the participant cities place them in different time zones (Spain in central European standard time (CET), Estonia in Eastern European standard time (EET), and Iceland in Greenwich mean time (GMT)). Based on the above and taking into account the multiple sleep quantity or quality deficit disorders as well as the lack of updated research on the existence (or not) of differences between sleep pattern of adolescents in different European, geographical time zones, the objective of the current exploratory research was to compare and analyze the perceived quality and duration of sleep in school adolescents living in cities that belongs to Mediterranean (Seville, Spain), Nordic (Reykjavik, Iceland) and Baltic (Tartu, Estonia) regions.

## 2. Materials and Methods

### 2.1. Study and Sample Design

This is cross-sectional, descriptive and quantitative study. We recruited a total of 1717 students between 13 to 16 years of age (52% boys and 48% girls) enrolled in public and private secondary schools in Seville (Spain), Reykjavik (Iceland) and Tartu (Estonia). For the purposes of the statistical analyses, a 95% confidence interval and a 5% margin of error were applied. The sample selection was done by convenience. The number of participating subjects and their different ages, both in total and for each participating city, are summarized in Table 1.

For our study, we recruited only the adolescents, who have delivered the informed consent document, which was signed by their parents/guardians. Verbal consent was given by the participants and they were informed that they could leave the investigation at any time. Finally, we only recruited adolescents who regularly attended school and took part in the physical education classes without any type of physical limitation. 

### 2.2. Instruments

The Pittsburgh Sleep Quality Index (PSQI) was used to assess the quality of sleep in a 1-month time period. Developed and validated by researchers at the University of Pittsburgh, PSQI is a standardized sleep questionnaire, aimed to be used by medical doctors and researchers. The questionnaire was handed to all the participants with statements in their mother tongue as well in English. For Spanish participants, we used the adapted Spanish version [37]. For the Icelandic and Estonian versions, parallel back-translation was used [38,39,40]. PSQI is widely used in the adolescent populations as it measures different components of sleep quality as subjective sleep quality, sleep disturbances, sleep latency, sleep duration, habitual sleep efficiency, use of sleep medications and daytime dysfunction [41,42,43,44]. The questionnaire consists of 19 items, which are grouped into 7 components that generate a final score. An overall score of 5 or more indicates poor sleep quality. To allow for comparison with similar studies, good quality of sleep would be exemplified as feeling rested and restored upon waking, low or non-existent number of awakenings at night and low or non-existent problems when falling asleep [45].

### 2.3. Procedures

All participants completed the PSQI questionnaire during their physical education classes and in the presence of the main researcher. This allowed the research team to answer any questions or address any problems related to the questionnaire, on the spot. Taking into account that hours of daylight directly influence the quality and duration of sleep, the duration and quality of sleep being lower with increasing exposure [46,47], data collection was planned and carried out using same daily light time in all three participating cities. Questionnaires were administered to the Icelandic and Estonian participants between October and mid-November, while for the Spanish participants the process took place in the months corresponding to the autumn season (October-December). Thus, the hours of daylight in the three participating cities were maintained between 9.5 and 10 h.

### 2.4. Data Analysis

Quantitative variables are presented as mean (M) ± standard deviation (SD). The normality of the variables was confirmed by Kolmogorov-Smirnoff test while homoskedasticity was assessed by using Levene’s test. For the analysis of the differences in sleep, between boys and girls in the three countries, a multivariate analysis of variance (MANOVA) was performed, including sleep duration (minutes) and total PSQI score as dependent variables, and with sex and the city used as independent variables. Age, height, and weight were also included as covariables. When statistically significant differences were detected, a Bonferroni post-hoc test was undertaken. Given that statistically significant sex differences were detected, a two-factor analysis of variance (ANOVA) for boys and girls including city and age (13, 14, 15 or 16 years) as independent variables was subsequently performed. Effect size (ES) was calculated using partial eta squared ($\eta_{p}^{2}$) considering < 0.25 as small, 0.26–0.63 as medium and >0.63 as large. In pairwise comparisons, Cohen’s d was calculated with values < 0.2, 0.2–0.5, 0.5–0.8 and >0.8 considered trivial, small, moderate and large, respectively. Additionally, the effect of sex and city was analyzed by Chi-square test and the probability of having a low quantity or poor quality of sleep (total PSQI score ≥ 5) was calculated by Odds Ratio (OR). The level of statistical significance was set as *p* < 0.05. The SPSS statistical package (version 18.0, SPSS Inc., Chicago, IL, USA) was used for all statistical analyses.

## 3. Results

### 3.1. Sleep Duration and Sleep Quality in Boys and Girls

The analysis of the participants that slept less than the recommended hours (8–10 h) revealed no statistical differences in the adolescents groups based in different countries (*p* = 0.095), with 67% in adolescents in Seville (615/917), 67% in Reykjavik (258/387) and 73% in Tartu (300/413) being found to have insufficient sleep duration. Similarly, no statistically significant differences were observed between boys and girls, with values being 68% and 69% for boys and girls, respectively (*p* = 0.716). The non-influence of age for sex (*p* = 0.814), city (*p* = 0.10) and the interaction (*p* = 0.186) were calculated. 

Regarding sleep duration (see Table 2), statistically significant differences were observed in the city·sex interaction (*p* < 0.001; $\eta_{p}^{2}$ = 0.014). Thus, girls from Sevilla slept 21 min longer than girls from Tartu (*p* < 0.001; d = 0.136) and a tendency towards significance with respect for those from Reykjavik was observed (*p* = 0.095; d =0.14). Boys from Tartu slept approximately 13 min more than those from Seville (*p* = 0.002; d = 0.21). 

Regarding the quality of sleep, a significant difference for cities was reported (*p* < 0.001; $\eta_{p}^{2}$ = 0.011) with a lower PSQI index in Reykjavik (4.35 ± 0.13) compared with Seville (5.11 ± 0.09; *p* < 0.001; d = 7.35) and Tartu (4.98 ± 0.13; *p* = 0.023; d = 4.85). Lower sleep quality was observed for girls in comparison to boys (total sample 5.10 ± 2.55 vs. 4.45 ± 2.38; *p* < 0.001; $\eta_{p}^{2}=0.013$). Boys from Seville presented lower sleep quality compared to those from Reykjavik (*p* < 0.001; d = 0.30), while boys from Tartu presented poorer sleep quality than Reykjavík boys (*p* = 0.021; d = 0.24). Girls from Seville had lower quality of sleep than those from Reykjavik (*p* = 0.014; d = 0.23). In addition, girls from Seville and Reykjavik presented inferior sleep quality compared to boys (*p* < 0.01).

### 3.2. Sleep Duration and Quality in Boys and Girls by Age

When analyzing the amount of sleep in the different age groups (13, 14, 15 and 16 years, see Table 3) different statistical differences were observed between cities (*p* = 0.007; $\eta_{p}^{2}$ = 0.011) and the interaction city·age (*p* < 0.001; $\eta_{p}^{2}$ = 0.027) in boys. Thirteen-year-old boys from Seville slept 32 min less than those from Reykjavik (*p* = 0.012; d = 0.51) and 38 min shorter than boys from Tartu (*p* = 0.001; d = 0.69) while 14-year-old boys in Tartu slept around 29 min longer than boys in Seville (*p* = 0.005; d = 0.49) and Reykjavik (*p* = 0.044; d = 0.51). In Seville, 15-year-old boys slept 34 min more than 13-year-olds (*p* < 0.001; d = 0.57) and 26 min more than 16-year-old boys (*p* = 0.013; d = 0.42) whereas in Tartu 16-year-old boys slept 30 min less than 13-year-olds (*p* = 0.041; d = 0.56) and 37 min less than their 14-year-old counterparts (*p* = 0.003; d = 0.69).

In relation to the amount of sleep among age groups (13, 14, 15 and 16 years), statistically significant differences were observed between cities for girls (*p* < 0.001; $\eta_{p}^{2}$ = 0.017), without any difference between ages (p = 0.061; $\eta_{p}^{2}$ = 0.009) or the city·age interaction (*p* = 0.272; $\eta_{p}^{2}$ = 0.009), although 16-year-old girls from Tartu slept 36 min less than those from Reykjavik (*p* = 0.006; d = 0.64). 

Regarding the quality of sleep, differences between cities (*p* < 0.001; $\eta_{p}^{2}$ = 0.017) and ages (*p* < 0.001; $\eta_{p}^{2}$ = 0.021) were detected, but not for the interaction city·age (*p* = 0.336; $\eta_{p}^{2}$ = 0.008). Thus, 16-year-old boys had lower quality of sleep compared than 13-year-old boys (*p* = 0.001; d = 5.29) and lower than 14-year-olds (*p* = 0.002; d = 5.16). Among the 14-year-old participants, boys from Seville had poorer quality of sleep than Reykjavik (*p* = 0.013; d = 0.33) and less than Tartu (*p* = 0.023; d =0.36). In the city of Tartu, 16-year-old boys had poorer quality of sleep than those of 13-year-olds (*p* = 0.012; d =0.66) and less than 14-year-olds (*p* = 0.002; d = 0.80).

Concerning girls, differences between cities (*p* = 0.005; $\eta_{p}^{2}$ = 0.013) and ages (*p* < 0.001; $\eta_{p}^{2}$ = 0.050) were reported, but not for the city·age interaction (*p* = 0.5; $\eta_{p}^{2}$ = 0.006). Sixteen-year-old girls presented poorer quality of sleep than 13-year-old (*p* < 0.001; d = 7.8), 14-year-old (*p* < 0.001; d =1.05) and 15-year-old girls (*p* < 0.001; d = 5.99). In the city of Seville, 15-year-old girls presented lower quality of sleep compared to those from Reykjavik (*p* = 0.014; d = 0.49). When comparing girls from Seville by age, 16-year-old girls had a sleep quality index lower than 13 years (*p* < 0.001; d = 0.62) and 14 years (*p* < 0.001; d = 0.49) and in those of 15 years with respect to those of 13 years (*p* = 0.008; d = 0.40). In Tartu, 16-year-old girls presented poorer quality of sleep than 13 (*p* = 0.018; d = 1.02), 14 (*p* < 0.001; d = 0.51) and 15-year-old girls (*p* = 0.018; d = 0.67).

### 3.3. Probability of Having Poor Sleep Quality by City and Sex

Regarding the quality of sleep, statistically significant differences were detected between the three cities (*p* = 0.005) (see Table 4). More specifically, adolescents in Seville and Tartu were more likely to have poor sleep quality than adolescents in Reykjavik (OR = 1.122 (1.07–1.40); OR = 1.20 (1.05–1.37)). In relation to the “by sex” analysis, no statistically significant differences were detected, but a trend towards statistical significance was observed for both boys (*p* = 0.095) and girls (*p* = 0.060). For example, we detected higher probabilities of suffering poor sleep quality for boys from Seville, in comparison to those from Reykjavik (OR = 1.22 (1.00–1.5038)) and, girls in Seville and Tartu in comparison to Reykjavik (OR = 1.21 (1.01–1.45); OR = 1.25 (1.01–1.54)) (see Table 4). 

On the other hand, the probability of having low quality sleep in girls was significantly higher in comparison to boys in the all three cities (*p* < 0.001; OR = 1.20 (1.09–1.33)), i.e., Seville (*p* = 0.010; OR = 1.19 (1.04–1.35)) and Tartu (*p* = 0.047; OR = 1.26 (1.01–1.58)) (see Table 5).

## 4. Discussion

The current study suggests that the majority of the participating adolescents from the three European countries/cities (Seville, Reykjavik and Tartu) did not meet recommendations for sufficient sleep hours per night for their age group. Additionally, we did not find any statistically significant differences by sex, regarding the insufficient sleep duration (i.e., 68% for boys and 69% for girls). In relation to the PSQI index of subjective sleep quality, 49% of the adolescents participating in our study (44% for boys and 53% for girls) showed poor quality of sleep. Contrary to the duration of sleep, statistically significant sex differences were found in sleep quality, with boys perceiving their sleep to be of better quality than girls. Higher probabilities of having low sleep quality were found in Seville and Tartu in comparison to Reykjavik and the probabilities of having a low-quality sleep in girls were significantly higher than for boys, in all three cities.

### 4.1. Sleep Duration

As previously discussed, the majority of the adolescents in all three cities did not reach the minimum recommended sleep duration for their age group [23]. This finding is in line with the results found in studies measuring sleep duration in each of the cities studied [48,49,50,51], as well as those found in a study that measured sleep duration of 220,000 European and North American adolescents [52]. 

Former research has been inconclusive regarding sex differences in sleep duration. For example, some studies report that girls have a longer duration of sleep [53], others report that boys do [51,54], while some suggest a similarity in duration between sexes [55]. Our results confirm these contradicting findings, as there was no uniformity for each sex in the different participating countries/cities. Exploring the reasons behind the low sleep duration can be equally challenging: A low duration among boys may be associated with a change in the maturational phase of development induced by testosterone [56], whose hormonal environment predisposes them to a late bedtime. Nevertheless, boys must get up at the same time as girls to fulfill their social and school commitments. For girls, reasons might also be of similar nature: factors such as increased stress and different hormonal changes typical of adolescence, as well as discomfort derived from the female menstrual cycle have been proposed as possible causes of low sleep duration [57]. Future research should focus on the origins of these differences and clarify reasons, allowing for the improvement of sleep disorder support in this age group.

When observing the city·sex interaction, significant differences were only detected between girls from Seville and Tartu. Previously published work suggests that healthier diet patterns can promote better sleep in adolescents [58,59]. Furthermore, greater adherence to the Mediterranean Diet has been associated with better sleep patterns (duration and quality) [27,60] as a consequence of the effect of different nutrients [61,62]. Therefore, the observed differences between the Seville and Tartu cohorts can be potentially attributed to the (expected) greater adherence to the Mediterranean Diet in girls from Seville compared to those from Tartu [58,59], although this would require further work to confirm.

Unlike previous reports on adolescent sleep duration, where less amount of sleep was found with increasing age [51,63,64], the age of the participants in the present study was not a determining factor that influences the duration. This is despite the fact that we found a non-statistically significant decrease in the duration of sleep, as the age of the subjects of the populations of Seville and Reykjavik increases. Prior findings show that as adolescents get older, their use of electronic devices and their time spent on screens increases, consequently reducing the duration of their sleep [65,66,67]. 

When observing the city·age interaction, it can be observed that there were significant differences between the shorter sleep duration of the 13-year-old boys from Seville compared to the boys from Reykjavik and Tartu and, for the 14-year-old boys from Seville compared to their counterparts from Tartu. A possible explanation for these results can be found in the lower levels of health-related physical fitness and higher levels of body composition of adolescents from Seville [58], when compared with Reykjavik and Tartu adolescents [59,68,69]. It is known that daily physical activity practice (and the consequent increase of the fitness level) is directly related to ease of falling asleep, thus achieving a longer duration [70,71,72,73]. 

Finally, the 16-year-old girls from Reykjavik reported significantly longer sleep duration than girls from Tartu. As previously discussed, a possible cause of the longer duration could be a higher Mediterranean diet adherence for the girls from Reykjavik compared to that observed in girls from Tartu [50,60].

### 4.2. Sleep Quality

In relation to sleep quality, more adolescents from Seville and Tartu (51%) reported poor perceived sleep quality (PSQI ≥ 5) than did those from Reykjavik (41%). Our data agrees with similar studies in the participating cities [64,74,75,76,77], as well as those reported in a longitudinal study that examined trends in the difficulty of sleeping in adolescents over 12 years [78].

When analyzing the perceived quality of sleep according to sex, boys from all countries/cities presented better PSQI index than girls, showing statistically significant differences in the cities of Seville and Reykjavik. This could be due to the fact that boys have higher rates of PA practice and physical exercise than girls at these ages [79], influencing not only the duration of sleep but the quality as well [80,81]. Another factor that could influence the difference in perceived quality, is the intake of sugary and stimulatory beverages. Although this increased consumption is now quite common among the whole European adolescent population [82], it is the girls who have the highest consumption [83,84,85], making it difficult for them to fall asleep during adolescence.

When comparing cities, a significantly higher PSQI index was obtained by participants from Seville in comparison to those from Reykjavik. These scores could be justified by recent studies, in which the subjects from Seville had a higher % of body fat compared to those from Reykjavik [58,69], which could affect their sleep quality [73,86,87]. Furthermore, the different meal times (especially in regards to dinner) characteristic of the Mediterranean population (<3 h before sleeping) could contribute to poor sleep quality due to gastrointestinal upset, heartburn and reflux [35]. On the other hand, higher PSQI scores obtained by boys from Tartu, compared to those from Reykjavik, could be explained by lower consumption of food and supplements rich in vitamin D. This low intake is associated with episodes of drowsiness during the day, longer sleep latency and shorter sleep duration, which would directly affect the perceived quality of sleep [88]. Nevertheless, these hypotheses should be studied in greater depth. 

When analyzing the PSQI index of sleep quality according to the age of the participants, it can be seen that in both boys and girls the index grows as their age increases. This is consistent with recent research carried out on the European adolescent population and reflects the worrying prevalence of poor sleep quality in this age group [78,89]. One of the reasons that can lead to a worse sleep quality as age increases may be the exposure to screens, since the older the subjects, the longer the time of use of electronic devices [70,90]. In addition, the negative influence is higher when close to bedtime, presenting higher levels of sleep latency and difficulties falling asleep [70,90]. 

Statistically significant differences were detected when comparing the quality of sleep in the three cities. Higher probabilities of having a low quality of sleep were found in Seville and Tartu in comparison to Reykjavik. In addition, girls present greater probabilities of poor sleep quality in the total of the three cities, as well as in Seville and Tartu independently. These results reflect how sex and country of origin are determining factors in their sleep quality. This data is similar to that previously found for sex and the region or country of origin [91]. Physiological [57], psychological [92] and social [93] differences between sex, as well as cultural differences [66,94] between different regions or countries could explain this. It is important to conduct further research to clarify the causes and (importantly) consequences, of these two factors, that is, the relationship between them and the quality/quantity of sleep (i.e., sedentary lifestyle, physical inactivity, screen exposure, poor diet or inadequate eating behaviors, stress, etc.). Moreover, this could be crucial when it comes to addressing public health policy and recommendations, as well as educating adolescents about good sleep hygiene. Additionally, it is of vital importance to limit their time of exposure to screens, electronic devices and, the intake of products that may alter consciousness and imply an increase of arousal before bedtime. 

Recently, a systematic review on cross-cultural comparative sleep in young populations has analyzed the roles of cultural factors [95]. The cross-cultural differences in sleep duration and quality may be explained by factors such as school start times [96], extracurricular school sports, homework, part-time work in older adolescents [97] and lack of parental limit-setting around bedtimes [98]. Other factors, such as diet or body fat percentage, may also contribute to sleep differences [99]. Moreover, geographical locations also seem to have an impact on sleep duration. Young populations in Northern Europe show a significantly longer nocturnal sleep duration to those in Southern (i.e., Spain) and Eastern (i.e., Estonia) Europe. The cultural preference for longer evenings in Southern European countries is negatively associated with sleep duration. Late dinner and the participation of children and adolescents in late evening social life explain different sleep patterns [95]. Iceland’s unique geographical location (latitude 64–66° North) results in a wide variation in daylight hours, 4–21 h between winter and summer months. A study using questionnaires and sleep diaries found that Icelandic youth had shorter sleep duration than their European peers [100]. Reduced daylight exposure in Northern European countries due to change of season has also been shown to prolong the onset of sleep [101].

This study has a number of limitations. Firstly, PSQI data collection was self-reported: this could lead to reporting error and memory bias. Nevertheless, it is a validated means of data collection of this type of data, with a relatively short participant burden. Secondly, our findings cannot be extended to the entire school population in the three countries that data was collected; however, the characteristics of the participants give us confidence that they represent a large proportion of the adolescent population in these cities. Thirdly, the implemented cross-sectional design does not allow us to infer the causal direction of our predictions. However, our findings can be used as valuable indicators that could form the basis for future research. Finally, and despite having adolescent populations from three European countries belonging to different geographical regions, it was not possible to analyze variables related to cultural aspects that could determine patterns of sleep duration and quality. Nevertheless, our work could be the starting point for subsequent studies looking at socio-cultural aspects and health habits of the population studied.

## 5. Conclusions

The current study showed that most of the adolescent participants had poor sleep quality, as they did not meet the recommended sleep duration per night for their age group. Although no statistically significant differences were found by sex concerning sleep duration, they did exist for sleep quality. Thus, better-perceived sleep quality was found in boys than in girls, who had higher probabilities of poor sleep quality. Moreover, these results are of particular concern for participants from Seville and Tartu, where the probability of poor sleep quality was found to be higher than in Reykjavik. Finally, sex and age are shown to be determining factors that can considerably modify sleep quality results. In light of our results, it is very important to advise and educate adolescents about the need of improving the quality and duration of their sleep, focusing on practical ways to achieve this.

## Figures and Tables

**Table 1:** Participants from the three cities by sex.

	Total (N = 1717)		Seville (917/1717)		Reykjavik (387/1717)		Tartu (413/1717)	
	**Boys**	**Girls**	**Boys**	**Girls**	**Boys**	**Girls**	**Boys**	**Girls**
	52%	48%	50%	50%	54%	46%	56%	44%
**13 years**	23%	22%	25%	24%	20%	24%	21%	16%
**14 years**	27%	27%	28%	29%	24%	24%	27%	23%
**15 years**	23%	26%	25%	26%	23%	24%	21%	30%
**16 years**	27%	25%	22%	21%	33%	28%	31%	31%

**Table 2:** Sleep duration and sleep quality in boys and girls in the three cities.

Variable	Sex	Seville	Reykjavik	Tartu
**Sleep duration (min.)**	Boys	439 ± 63 *	451 ± 66	452 ± 52 *
	Girls	450 ± 62 ^A^	442 ± 56	429 ± 53
**Sleep quality (index)**	Boys	4.79 ± 2.78 *^, B^	4.02 ± 2.10 *^, C^	4.54 ± 2.26
	Girls	5.41 ± 2.96 ^B^	4.78 ± 2.34 *	5.13 ± 2.35

* Statistical difference between boys and girls in the same city; ^A^ Significance of the difference between Sevilla and Tartu; ^B^ Significance of the difference between Sevilla and Reykjavik; ^C^ Significance of the difference between Reykjavik and Tartu.

**Table 3:** Sleeping duration and quality in boys and girls by age in the three cities.

Variable	Sex	Age	Seville	Reykjavik	Tartu
**Sleep duration (minutes)**	Boys	13 years	424 ± 59 ^A^	456 ± 69	462 ± 48
		14 years	440 ± 66 ^C^	441 ± 64 ^D^	469 ± 46
		15 years	458 ± 60 *	452 ± 71	444 ± 44
		16 years	432 ± 60 Φ	452 ± 62	432 ± 58 *, λ
	Girls	13 years	448 ± 57	440 ± 54	432 ± 46
		14 years	459 ± 64	440 ± 61	436 ± 63
		15 years	457 ± 64	440 ± 55	440 ± 39
		16 years	433 ± 59	447 ± 55 ^D^	411 ± 55
**Sleep quality (index)**	Boys	13 years	4.33 ± 2.54	3.86 ± 2.13	4.00 ± 2.52
		14 years	4.85 ± 3.07 ^A^	3.69 ± 2.09	3.84 ± 2.12
		15 years	4.88 ± 2.70	3.96 ± 2.07	4.69 ± 2.32
		16 years	5.11 ± 2.71	4.42 ± 2.08	5.41 ± 1.84 *, λ
	Girls	13 years	4.60 ± 2.66	4.38 ± 2.23	4.55 ± 2.26
		14 years	5.04 ± 2.57	4.51 ± 2.33	4.21 ± 1.84
		15 years	5.74 ± 3.024 *^, A^	4.40 ± 1.75	4.85 ± 2.27
		16 years	6.46 ± 3.37 *, λ	5.66 ± 3.26	6.38 ± 2.36 *, λ, Φ

^A^ Significance of the difference between Sevilla vs. Reykjavik and Tartu; ^C^ Significance of the difference between Sevilla and Tartu; ^D^ Significance of the difference between Reykjavik and Tartu; * Statistical difference for 13 years; *λ* Statistical difference for 14 years; Φ Statistical difference for 15 years.

**Table 4:** Probability of having poor sleep quality in boys and girls by cities.

Sex	PSQI < 5 Points			PSQI ≥ 5 Points			*p*-Value	OR _Seville vs. Reykjavik_	OR _Seville vs. Tartu_	OR _Tartu vs. Reykjavik_
	Seville	Reykjavik	Tartu	Seville	Reykjavik	Tartu				
**Total**	49%	59%	49%	51%	41%	51%	0.005 *	1.22 (1.07−1.40)	1.00 (0.93−1.07)	1.20 (1.05−1.37)
**Boys**	54%	62%	54%	46%	38%	46%	0.095	1.22 (1.00−1.50)	0.99 (0.90−1.11)	1.18 (0.99−1.40)
**Girls**	45%	55%	43%	55%	45%	57%	0.060	1.21 (1.01−1.45)	0.98 (0.89−1.08)	1.25 (1.01−1.54)

* Statistically significant differences at *p* < 0.05. PSQI = Pittsburgh Sleep Quality Index; PSQI < 5 points = good quality; PSQI ≥ 5 points = poor quality (high sleep latency, sleep disturbances and daytime dysfunction).

**Table 5:** Probability of having poor sleep quality in the three cities by sex.

City	PSQI < 5 Points		PSQI ≥ 5 Points		*p*-Value	OR _Girls vs. Boys_
	Boys	Girls	Boys	Girls		
**Total**	56%	47%	44%	53%	<0.001 *	1.20 (1.09–1.33)
**Seville**	54%	45%	46%	55%	0.010 *	1.19 (1.04–1.35)
**Reykjavik**	62%	54%	38%	46%	0.181	1.17 (0.95–1.45)
**Tartu**	54%	43%	46%	57%	0.047 *	1.26 (1.01–1.58)

* Statistically significant differences at *p* < 0.05. PSQI = Pittsburgh Sleep Quality Index; PSQI < 5 points = good quality; PSQI ≥ 5 points = poor quality (high sleep latency, sleep disturbances and daytime dysfunction).

## Data Availability

The data presented in this study are available on request from the corresponding author. The data are not publicly available due to privacy reasons.
